# Resistant starches from dietary pulses improve neurocognitive health via gut-microbiome-brain axis in aged mice

**DOI:** 10.3389/fnut.2024.1322201

**Published:** 2024-01-24

**Authors:** Saurabh Kadyan, Gwoncheol Park, Nathaniel Hochuli, Katelyn Miller, Bo Wang, Ravinder Nagpal

**Affiliations:** ^1^The Gut Biome Lab, Department of Health, Nutrition, and Food Sciences, College of Education, Health, and Human Sciences, Florida State University, Tallahassee, FL, United States; ^2^Department of Biomedical and Chemical Engineering and Sciences, Florida Institute of Technology, Melbourne, FL, United States

**Keywords:** microbiota, plant polysaccharides, metabolic diseases, aging, neuroinflammation

## Abstract

**Introduction:**

Cognitive decline is a common consequence of aging. Dietary patterns that lack fibers and are high in saturated fats worsen cognitive impairment by triggering pro-inflammatory pathways and metabolic dysfunctions. Emerging evidence highlights the neurocognitive benefits of fiber-rich diets and the crucial role of gut-microbiome-brain signaling. However, the mechanisms of this diet-microbiome-brain regulation remain largely unclear.

**Methods:**

Accordingly, we herein investigated the unexplored neuroprotective mechanisms of dietary pulses-derived resistant starch (RS) in improving aging-associated neurocognitive function in an aged (60-weeks old) murine model carrying a human microbiome.

**Results and discussion:**

Following 20-weeks dietary regimen which included a western-style diet without (control; CTL) or with 5% w/w fortification with RS from pinto beans (PTB), black-eyed-peas (BEP), lentils (LEN), chickpeas (CKP), or inulin fiber (INU), we find that RS, particularly from LEN, ameliorate the cognitive impairments induced by western diet. Mechanistically, RS-mediated improvements in neurocognitive assessments are attributed to positive remodeling of the gut microbiome-metabolome arrays, which include increased short-chain fatty acids and reduced branched-chain amino acids levels. This microbiome-metabolite-brain signaling cascade represses neuroinflammation, cellular senescence, and serum leptin/insulin levels, while enhancing lipid metabolism through improved hepatic function. Altogether, the data demonstrate the prebiotic effects of RS in improving neurocognitive function via modulating the gut-brain axis.

## 1 Introduction

Aging refers to the complex multifactorial process involving progressive decline in the homeostatic functioning of various organs and tissues throughout the lifespan (1). One of the prominent characteristics of the aging process is the neurocognitive decline, particularly in areas related to learning and memory impairments. Besides, cognitive deficits in older people can be exacerbated by various lifestyle- and environment-related risk factors, with diet being a particularly significant factor to consider (2). Ample evidence has indicated that poor dietary patterns, especially associated with western-style diets rich in fat and sugar, contributes to metabolic dysfunction which, in turn, impair cognition via multiple cellular signaling mechanisms (3). On the contrary, prudent/healthy dietary patterns rich in dietary fibers, omega-3 fatty acids, and polyphenols support cognitive function and mental health (4). Therefore, tailoring the nutritional needs of older adults aimed at improving brain health holds promise in preventing aging-associated cognitive decline.

The gut microbiome has recently emerged as a significant influencer of healthy aging, brain plasticity, and neurocognitive health due to its growing role in the regulation of gut-brain axis (5). The composition of gut microbial populations undergoes changes in response to consumed food components, which subsequently communicates with the brain through interrelated and complex mechanisms. These mechanisms of bidirectional crosstalk between gut and brain involve neuronal innervation, hepatic metabolism, immunomodulation, enteroendocrine, and microbial metabolite signaling pathways (6). In line with these mechanisms, the western diet-induced deteriorations in the central nervous system (CNS) have been closely linked to diet-induced gut dysbiosis, impaired intestinal epithelial integrity (“leaky gut”), and aggravated local and systemic inflammation. In contrast, fiber-rich diets promote gut homeostasis, epithelial barrier integrity, balanced inflammatory tone, thereby leading to normal CNS functions (7). Moreover, the intake of dietary fibers not only alleviates cognitive dysfunction within the current generation but may also exhibit beneficial effects at transgenerational levels (8). Production of short-chain fatty acids (SCFAs) by the gut microbial metabolism of dietary fibers are reported for their benign effects on brain functions via amelioration of neuroinflammation, enhancement of synaptic plasticity via induction of gut-hormones, microglia maturation and function (4).

**Figure 1:**
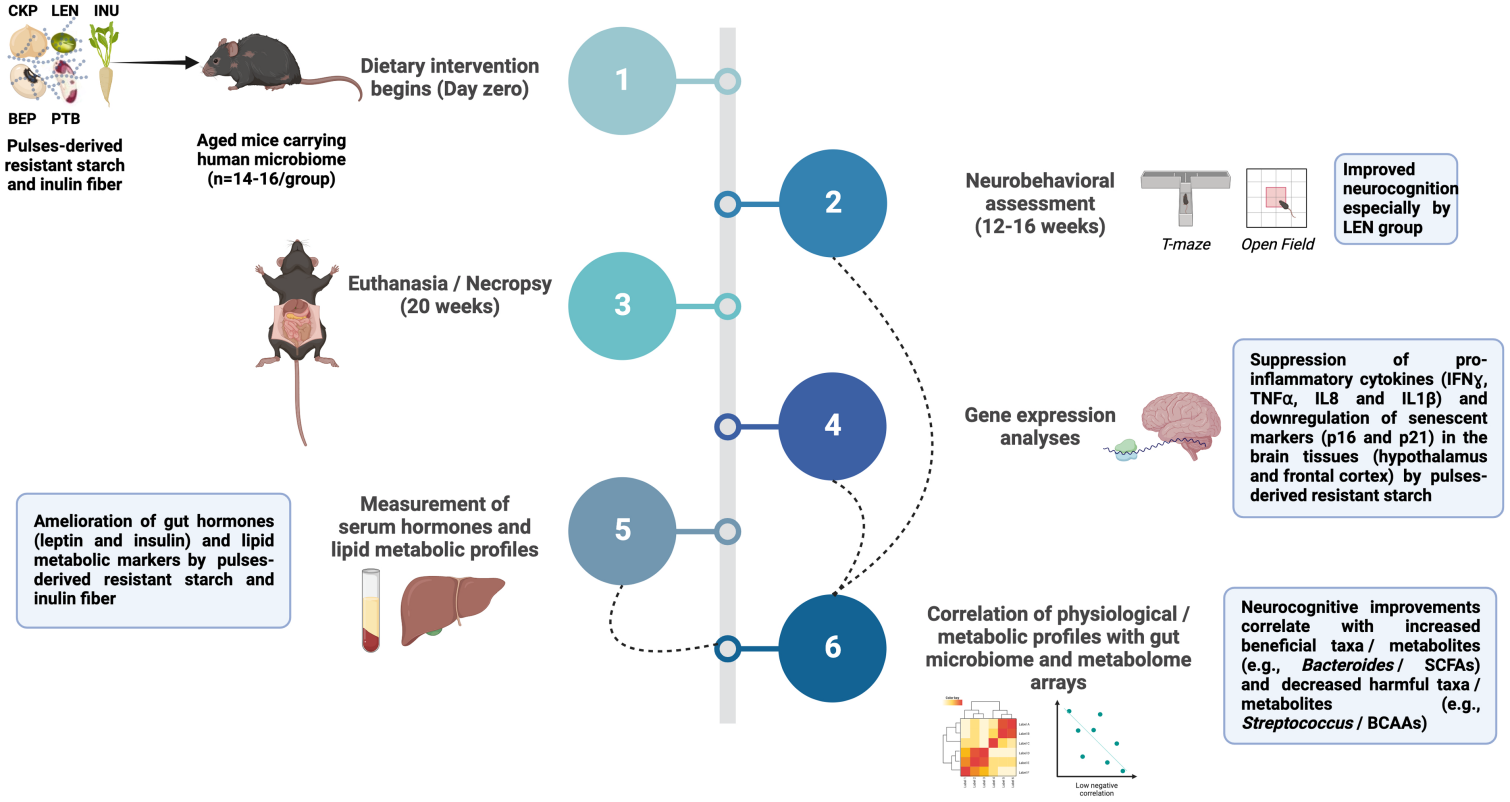
Summary of the study workflow and key findings. Treatment groups included western-style high-fat diet supplemented with 5% w/w with inulin fiber (INU), resistant starch derived from chickpea (CKP), lentils (LEN), black-eyed peas (BEP), and pinto beans (PTB); SCFA, Short-chain fatty acids; BCAAs, Branched-chain amino acids; IFNɣ, Interferon gamma; TNFα, Tumor necrosis factor alpha; IL, Interleukin.

An important dietary fiber widely present in plant-based foods is resistant starch (RS), which possesses the potential to modulate the gut-brain axis due to its characteristics that make it accessible for fermentation by the microbiota in the lower gut leading to the production of SCFAs and other beneficial metabolites and neurotransmitters. Several studies have evidenced the promising potential of RS in improving gut microbiome and peripheral health outcomes (9, 10), but our understanding of its modulatory effects on the gut-brain axis remains limited (11–14). Dietary pulses (e.g., beans, legumes, and peas), which are a significant source of RS, have long been associated with improved cardio-metabolic and cognitive health (15, 16). Despite this, the neuroprotective effects of dietary pulses-derived resistant starches in promoting neuronal functions via modulation of gut microbiome remain largely unexamined. Our recent studies have demonstrated that RS extracted from four different pulse varieties differently modulate gut microbiome and metabolome while exerting positive systemic effects such as attenuated post-prandial glycemia, improved gut barrier integrity and reduced intestinal inflammation in a “humanized” mouse model of aging (17, 18). Propelled by and building upon these findings, we herein aimed to further explore whether the RS-mediated intestinal remodeling affects cognitive functions via microbiota-gut-brain axis signaling mechanisms in the western-stye HFD-fed aged mice carrying human gut microbiome. We find that only LEN group (lentils-derived RS) exhibits superior neurocognitive performance in open-field and T-maze tests. However, RS intervention exerts positive effects on brain function by resolving neuroinflammation, suppressing cellular senescence, improving hepatic metabolism, and mitigating/ normalizing serum leptin and insulin levels. Further correlational analysis reveals valuable insights on the host-microbiota-metabolite crosstalk in fine tuning the above responses. The entire workflow of the study along with key findings was summarized in Figure 1. Taken together, these findings contribute to a mechanistic understanding of the mind-altering potential of pulses-derived RS, highlighting the involvement of immune, endocrine, and metabolic pathways within the microbiome-gut-brain axis.

## 2 Materials and methods

### 2.1 Production of RS from pulses

The production of resistant starch from pulse seeds was performed as per our previously described method (18, 19). In brief, 12 grams of starch were gelatinized in 240 mL of sodium phosphate buffer (pH 6.9) and cooled to 37°C. Subsequently, the mixture was incubated for 15 min in the presence of 2 mL of salivary amylase (Sigma-Aldrich). Continuous stirring was maintained during hydrolysis, while the pH was adjusted from 6.9 to 2.0 using 6 M HCl. Sequential addition of enzymes was then initiated for digestion: pepsin (37°C, pH 2.0, 30 min) followed by the addition of 4 mL of pancreatin (37°C, pH 6.9, 90 min). The hydrolyzed starch was subjected to dialysis (molecular weight cutoff: 6–8 kDa) for 36 h, and the remaining undigested starch was freeze-dried for 72 h.

### 2.2 Animal study design

The animal experimentation procedures followed our previously established protocol (18). Briefly, the native gut microbiota of 55-week-old C57BL/6 J mice was eliminated (gut depletion) through a four-day period of *ad-libitum* feeding with an antibiotic cocktail (ampicillin [1 g], metronidazole [1 g], neomycin [1 g], and vancomycin [0.5 g] per liter of drinking water), followed by the administration of four doses of oral gavage (gut cleansing) with polyethylene glycol (200 μL per dose; 425 g/L). Subsequently, fecal samples obtained from five human older adult donors (age 50–55 years) were pooled in equal proportion and transplanted into the mice gut according to our previously described method. Donors were chosen based on their adherence to an average American dietary pattern, characterized by none to rare consumption of pulses. They were apparently healthy, with no reported intestinal or digestive issues and no use of antibiotics for at least 6 months prior to sample collection. The mice were subsequently randomly divided into six groups (*n* = 14–16/group; 7–8 for each sex), before commencing a 20-week dietary intervention (refer Figure 1 for complete workflow). The groups included: CTL (control group on a western-style high-fat diet), four treatment groups with the CTL diet supplemented with resistant starch (5% w/w) derived from pinto beans (PTB), black-eyed peas (BEP), lentils (LEN), and chickpeas (CKP), respectively, and a reference group (INU) with 5% w/w inclusion of inulin in the CTL diet. Mice were assessed for the neurobehavior parameters starting 12-weeks of dietary intervention. At the study endpoint, each mouse was positioned in the induction chamber of the precision vaporizer and anesthetized with isoflurane (3% in pure oxygen; flow rate – 1 L/min). After induction, the mouse was placed on nose cone in a dorsal position and was maintained on 1% isoflurane for intra-cardiac blood collection. Subsequently, the mouse was flipped into a sternal position and euthanized by cervical dislocation. Tissues and serum samples were collected in liquid nitrogen and stored immediately at −80°C for further analyses.

### 2.3 Neurocognitive tests

#### 2.3.1 Open-field test

The procedure was carried out in a dedicated procedure room within the vivarium facility to assess basic locomotor activity. Mice were gently placed at the center of a clean and sanitized box (40 × 40 cm^2^), and mice movements were video recorded for 5 min. The measures of behavior parameters such as mobility, total distance travelled, and percentage time spent in the center were assessed by using Ethovision XT software (Noldus) (20).

#### 2.3.2 Novel object test

The test was carried out in accordance with previously described protocol (20), without habituation phase. Briefly, two similar objects were placed in the chamber (same as open-field arena) and mice were allowed to explore them for 5 min. After 24 h, mice were re-tested for memory retention by placing two objects in the chamber in the same locations but replacing object 2 (familiar object: wooden cube) with object 3 (novel object: black rubber cylinder) and again scoring orientation for a 5 min interval. The time spent exploring the objects was assessed using Ethovision XT software when mouse directed its nose tip within 2 cm radius of objects. Memory retention was calculated using discrimination index: [(time spent exploring the new object) − (time spent exploring familiar object)]/(the total exploring time for all objects).

#### 2.3.3 Location memory test

The test was conducted as per previously described protocol (21), with some modifications. Briefly, four different objects were placed equidistant from each other in the open-field arena. Mice were gently placed at one end facing towards wall of arena and allowed to explore freely for 5 min. After 24 h, location of two adjacent objects were flipped and the spatial memory retention of mouse was assessed by videorecording for 5 min. The time spent exploring the objects was assessed using Ethovision XT when mouse directed its nose tip within 2 cm radius of objects. Memory retention was calculated using discrimination index: [(time spent exploring the relocated objects) − (the time spent exploring the familiar objects)]/(the total exploring time).

#### 2.3.4 T-maze spontaneous test

Spatial working memory of rodents was assessed using T-maze test as per previously described protocol (22). Briefly, mice were gently placed at the distal end of the start arm, with head facing towards the southern end of wall. Mice were allowed to move spontaneously, and their respective alterations (left or right) in the goal arms were recorded. Each mouse was tested for seven trials and apparatus was cleaned with 70% ethanol within each mouse. The percent alteration score as an index of working memory was calculated using formula: (total number of correct alternations/6) * 100.

### 2.4 Neuromuscular and motor function tests

The neurobehavioral coordination of mice was evaluated through three tests: grip strength test, rotarod test, and hanging wire test. A grip strength meter (World Precision Instruments) was used to measure forelimb grip strength. The forelimb grip strength of each animal was measured by placing its forelimbs on the brass pole and gently pulling its tail until the grip was released. This test was performed once per mouse, and the results from three separate trials were recorded and averaged to determine the forelimb grip strength (in mN) for each mouse. For the rotarod test, the mice underwent a training session lasting 5 min, during which they were placed on the Rotarod apparatus (Harvard Apparatus) rotating at a constant speed of 4 rpm. On the following day, the mice were tested by placing them on the apparatus and initiating rotation at 4 rpm, with subsequent increments of 1 rpm every 8 s. The time until each mouse fell from the rotarod was recorded, and each trial, with 5 min rest interval, was repeated three times for each mouse. Hanging wire test was performed as described by Hoffman et al. (23). Briefly, a circular wire (32 cm in length and 2.5 mm in diameter) was securely mounted on a stand, allowing free circular motion. The mice were positioned underneath the wire, ensuring that all four paws were in contact. Three separate hang times were recorded, with a 30 s rest period between each measurement. There was no restriction on the duration of each individual hanging period.

### 2.5 Gene expression analysis for tight-junctions, inflammatory, and senescence markers in the brain

Total RNA from the snap-frozen brain tissues (hypothalamus and frontal cortex) were isolated using RNeasy kit (Qiagen), followed by reverse transcription using the High-capacity cDNA reverse transcription kit (ThermoFisher). The mRNA expression of tight-junction proteins, including claudin-1 (CLDN1), claudin-5 (CLDN5), occluding (OCCL), zonulin-1 (ZO1), and JAM3 gene; inflammatory markers including interleukins (IL1β, IL6, IL8, IL10), interferon-gamma (IFNɣ), and tumor necrosis factor-alpha (TNF-α); and senescence markers (p16 an p21) were quantified using real-time qPCR (QuantStudio3, Applied Biosystems). The 18S gene was used as an internal housekeeping control. The results were expressed as ddCt method by normalizing against the 18S expression of the control group, as per our earlier report (11, 24). Additionally, colonic, and ileal tissues underwent the same assessment for senescence markers. The details of primers’ sequence were retrieved from previous studies (25–27); primer details are presented in Supplementary Table S1.

### 2.6 Measurement of serum hormones

ELISA kits were used to measure serum levels of leptin, insulin and glucagon according to the manufacturer’s instructions (AssayGenie, Ireland). All samples’ measurements were performed in duplicate.

### 2.7 Serum lipid profiles

The concentrations of circulating serum cholesterol (CHOL), triglycerides (TRIG), high-density lipoprotein cholesterol (HDL), low-density lipoprotein cholesterol (LDL), very low-density lipoprotein cholesterol (VLDL), non-HDL cholesterol, total cholesterol-to-HDL(TC/H) ratio, aspartate aminotransferase (AST), alanine aminotransferase (ALT), glucose (GLU) were determined using the Piccolo Xpress Chemistry Analyzer (Abaxis, United States) by utilizing a Piccolo Lipid Panel Plus Reagent Disc according to the manufacturer’s instructions.

### 2.8 Correlational analysis of gut microbiome-metabolome interaction with neurocognition, inflammation, endocrine, and hepatic metabolism

The analyses pertaining to the modulatory effects of these four RS’s on gut microbiome and metabolome have been described in our recent studies (17, 18). Downstream correlational analysis of the microbiome and metabolome signatures with the herein studied parameters, as well as data visualization, was performed using “R” and “Python” packages. Spearman correlation analysis was conducted to examine the relationships between behavior test results, ELISA measurements, and Piccolo assay outcomes with the respective genera and metabolites. Additionally, the same correlation analyses were performed to investigate the relationships among genes, genera, and metabolites in both the hypothalamus and frontal cortex. For the multiple comparison, *p*-values were corrected using Benjamini-Hochberg method. The results were visualized using either a heatmap or a circos plot. For the circos plot, it was constructed to depict the correlations among genes, genera, and metabolites, and it included only correlation results with metabolites or genera displaying correlational relationship with at least one of genes (ρ > 0.3).

### 2.9 Statistical analysis

All values are presented as mean ± standard error of the mean (SEM). Unless otherwise stated, statistical significance was assessed at *p* < 0.05. For the evaluation of behavioral tests, all mice (*N* = 6–8 per group per sex) were included, except for the novel object, location memory, and T-maze tests, where the sample size was *N* = 4 per group per sex. In measuring serum hormones and lipid profiles, two samples per cage were combined to create three sets per group per sex, which were subsequently analyzed. All statistical analyses were performed using SPSS version 29.0.1.0. Statistical significance for neurobehavioral, hormonal, and lipid profiles was evaluated using analysis of covariance (ANCOVA) following LSD post-hoc analysis for comparing each treatment group with control, after adjusting for sex. Linear regression was carried out to predict associations for novel object and location memory tests. Cohen’s d effect size, along with 95% confidence limits, was ascertained using Student’s *t*-test. Gene expression analysis involved a sample size of 6–8 samples per group per sex. To analyze non-parametrically distributed gene expression data, the Kruskal-Wallis test, followed by Dunn’s post-hoc analysis, was utilized to investigate statistical differences between each treatment group and the control group, considering both combined and individual sexes. Additionally, to assess the impact of various treatment groups and brain regions on gene expression fold change, a two-way analysis of variance (ANOVA) test was performed on rank-transformed gene expression results. The graphs were created using GraphPad Prism version 10.1.0. Data from both males and females were collectively analyzed and presented in the main text, while sex-specific data was visualized in Supplementary Figures.

## 3 Results

### 3.1 Effect of RS on neurobehavioral outcomes

**Figure 2:**
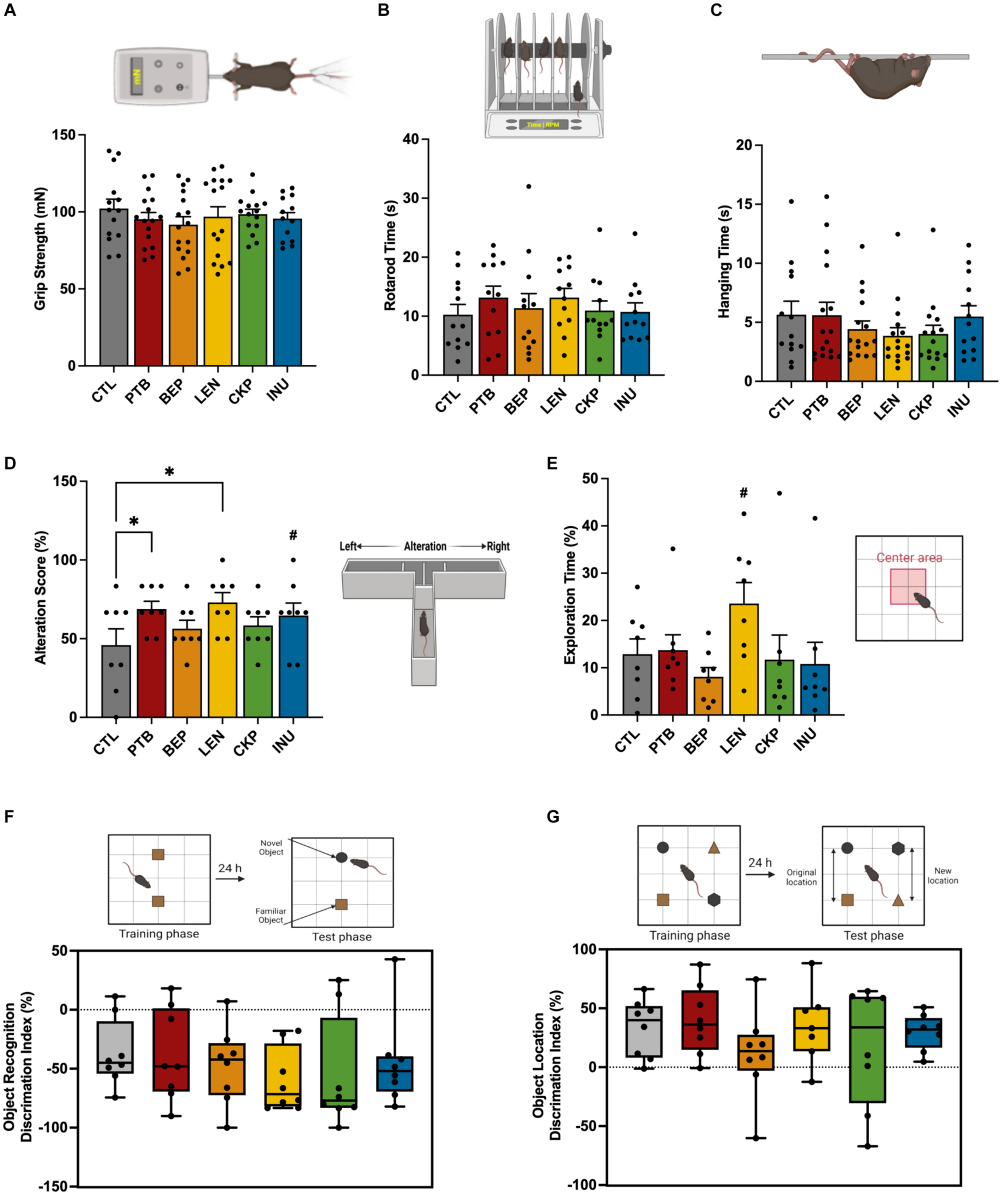
Effects of RS and inulin supplementation on neurobehavoiral outcomes in western-style HFD-fed aged mice: **(A)** Grip strength test, **(B)** Rotarod test, **(C)** Hanging wire test, **(D)** T-maze spontaneous test, **(E)** Open-field test, **(F)** Novel object test, and **(G)** Location memory test. CTL, control group; PTB, pinto beans; BEP, black-eyed-peas; LEN, lentils; CKP, chickpeas; INU, inulin. Data includes both genders; pairwise comparisons adjusted for sex. *p < 0.05, ^#^*p* < 0.1.

The effect of RS-supplemented westernized diet in improving the overall cognitive functions of aged mice was evaluated using a battery of neurobehavioral tests as summarized in Figure 2. There were no statistically significant differences in the neuromuscular coordination between RS and control groups during grip strength, hanging wire and rotarod tests (Figures 2A–C). However, there was a visible improvement in the spatial learning and memory of experimental mice against control as assessed via alteration score during T-maze test, which was significantly enhanced for LEN (72.92 ± 6.25%, *p* = 0.008) and PTB (68.75 ± 4.92%, *p* = 0.024) compared to CTL (45.83 + 10.32%) group (Figure 2D). Additionally, INU group also demonstrated slightly higher alteration score (64.58 ± 8.00%, *p* = 0.061) compared to CTL group. Among other neurocognitive tests, mice from LEN group spent nearly double the time exploring center (23.57 ± 4.43%, *p* = 0.058) compared to control group (12.87 ± 3.21%) in an open field test, while also revealing a relatively large effect size (*d* = −0.977, CI_95_ = [−2.005, 0.082]) (Figure 2E). Besides, there was no significant difference in the total distance traversed and mobility parameters among treatment and control groups during open field test, suggesting that they did not exert any influence on exploration times (Supplementary Figures S1A,B). The retention of memory for an object’s identity and its spatial location was assessed using widely applied novel object recognition and location memory tests (Figures 2F,G), respectively. Linear regression analysis, followed by ANCOVA, was employed to model the relationship between the time spent exploring new and old objects in the novel object test, and the time spent exploring familiar and relocated objects in the location memory test, across various diet groups (Supplementary Tables S2A,B). In the novel object test, the regression model demonstrated a good fit (*F* = 2.999, *p* < 0.05), explaining 31.9% of the variation in the time spent on new objects based on the time spent on familiar objects, various diet groups, and the interactions between them. However, only interaction between BEP group and time spent on familiar object found statistically significant (*p* = 0.003). Nevertheless, following adjustment for the time spent on familiar objects through ANCOVA, no statistically significant difference was observed among diet groups (*p* = 0.295) (Supplementary Table S2B). The negative discrimination index for novel object recognition, indicating more time spent on the familiar object, also exhibited a relatively larger effect in the LEN group (*d* = 0.815, CI95 = [−0.223, 1.826]), followed by a moderate effect in the CKP group (*d* = 0.482, CI95 = [−0.522, 1.469]), compared to the CTL group (Figure 2F). This observation also aligns with the regression model results, where the LEN and CKP groups exhibited a negative association with the time spent on the new object (Supplementary Table S2A).

For the location memory test, no statistically significant linear dependence of the mean of time spent on relocated objects on time spent on familiar objects across different diet groups was explained by the regression model (*F* = 1.726, *p* = 0.109, adjusted *R*^2^ = 15.1%) (Supplementary Table S2A). Further, ANCOVA analysis did not reveal any significant effect (*p* = 0.654) of the diet groups on time spent on relocated objects (Supplementary Table S2B). PTB group exhibited a slightly higher positive discrimination index compared to the CTL group; however, it showed a smaller overall effect (*d* = −0.246, CI95 = [−1.226, 0.742]) (Figure 2G). There were no notable sex-specific differences in these evaluated neurobehavior outcomes after comparing each treatment group with the control group (Supplementary Figures S1C–I). Overall, LEN group yielded consistent improvements in spatial memory recognition and exploratory behaviors in a humanized murine model of aging.

### 3.2 Effects of RS on tight-junctions, inflammation, and senescence markers in brain

**Figure 3:**
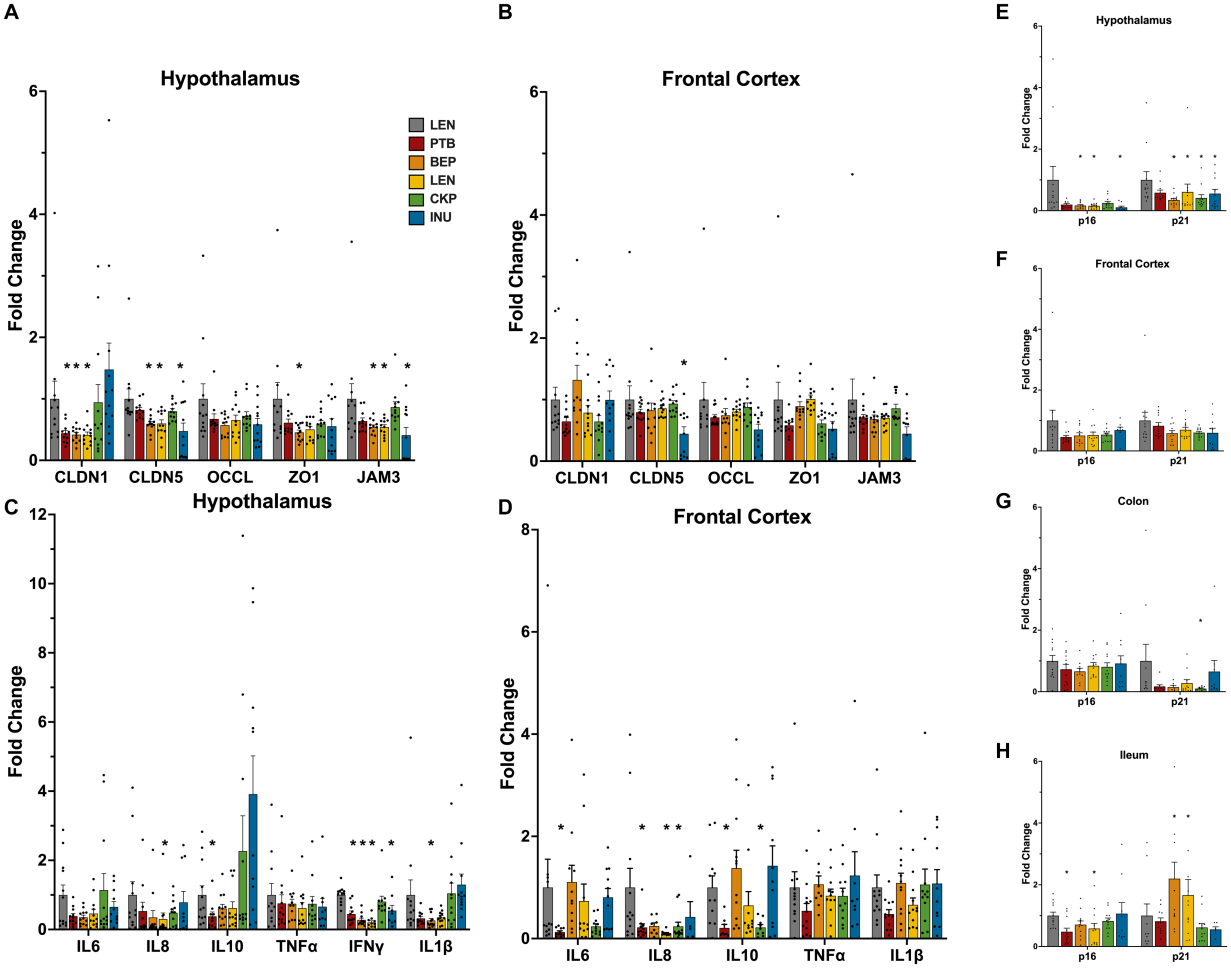
Modulatory effects of RS and inulin supplementation on the mRNA expression levels of tight-junction proteins, inflammation, and senescence markers in brain tissues: Tight-junction proteins expression in **(A)** hypothalamus, and **(B)** frontal cortex; Inflammatory markers expression in **(C)** hypothalamus, and **(D)** fronal cortex; Cellular senescence markers expression in **(E)** hypothalamus, **(F)** frontal cortex, **(G)** colon, and **(H)** ileum. CTL, control group; PTB, pinto beans; BEP, black-eyed-peas; LEN, lentils; CKP, chickpeas; INU, inulin. Data includes both genders. *p < 0.05.

The overall influence of RS in modulating the expression of tight-junction proteins (TJPs), inflammation and senescence markers in the hypothalamus and frontal cortex (FC) regions of brain are summarized in Figure 3. Additionally, a two-way ANOVA, conducted on the rank-transformed fold change data of tight junction proteins (TJPs), inflammatory markers, and senescence markers, revealed significant effects attributed to diet groups, brain regions, and the interaction between them (Supplementary Figure S2). Overall, all genes, except ZO1 and TNF⍺, were influenced by the different diet groups. Furthermore, the expression of all genes, excluding JAM3, IL6, IL8, and IL10, exhibited variations across different brain regions. Notably, the interaction between diet groups and brain regions specifically influenced the expression levels of ZO1, CLDN1, CLDN5, IL6, and IL1β.

Following the RS intervention, the alteration in the expression of TJPs was more prominent in hypothalamus. In some treatment groups, the expression of CLDN1, CLDN5 and JAM3 was found to be reduced (Figures 3A,B). The expression of the CLDN5 and JAM3 gene was significantly lower (*p* < 0.05) in the hypothalamus of the BEP, LEN, and INU groups, while CLDN1 expression reduced significantly (*p* < 0.05) in PTB, BEP and LEN groups (Figure 3A). Besides, the relative expression pattern of TJPs differed among sexes (Supplementary Figure S3A). Specifically, in the male hypothalamus, the expression of TJPs (CLDN5, OCCL, ZO1, and JAM3) significantly decreased in the BEP, LEN, and INU groups. In contrast, LEN, CKP and INU groups exhibited an upward trend in the expression of ZO1, JAM3 and OCCL genes in females (Supplementary Figure S3A). In the FC region overall, tight junction proteins (TJPs) were not significantly affected, except for a notable reduction (*p* < 0.05) in CLDN5 by the INU group and an enhanced expression of CLDN1 by the BEP group (Figure 3B). Interestingly, CKP and LEN exerted female-driven effect by significantly enhancing (*p* < 0.05) expression of CLDN5 and ZO1/JAM3, respectively (Supplementary Figure S3B).

The RS intervention also modulated the inflammatory tone, with a more positive effect observed in the hypothalamus relative to FC. All treatment groups except CKP significantly reduced (*p* < 0.05) the overall expression of IFNɣ in the hypothalamus (Figure 3C). Furthermore, such effects were also visible in males and females (Supplementary Figure S3C). We noted a significant downregulation of IL1β in the BEP group, as well as a reduction in IL8 in the LEN group (Figure 3C). These trends were also observed in the tissues of male subjects when analyzed separately (Supplementary Figure S3C). Albeit non-significant, the downregulatory effect of RSs on TNF⍺ and IL1β appeared more prominent in hypothalamus region of males than females (Supplementary Figure S3C). Overall, the expression of IL8 cytokine in the FC was significantly lower (*p* < 0.05) for the PTB, LEN, and CKP groups compared to CTL (Figure 3D). Besides, IL10 was also downregulated significantly (*p* < 0.05) by PTB and CKP groups. Similar profiles of inflammatory response in FC also revealed in males and females (Supplementary Figure S3D).

Importantly, in particular relevance to aging, the expression of senescent markers p16 and p21 in the brain tissues exhibited a reducing trend in treatment groups compared to control (Figures 3E,F). Besides, both were significantly downregulated (*p* < 0.05) by BEP, LEN, and INU groups in the hypothalamus (Figure 3E). Similar trends for these senescent genes were also observed separately in males and females (Supplementary Figures S3E,F). Additionally, we also observed expression of these markers in colonic and ileal tissues (Figures 3G,H). The expression of p21 in the colon was suppressed by all treatment groups, with the CKP group showing a significant reduction (Figure 3G). In the ileum, the expression of p16 was significantly reduced (*p* < 0.05) by PTB and LEN groups, while the expression of p21 increased in the BEP and LEN group (Figure 3H). Overall, RSs explicitly reduced the expression of senescence associated genes in different brain regions.

### 3.3 Effects of RS on the endocrine responses of glucose, appetite, and lipid metabolism

**Figure 4:**
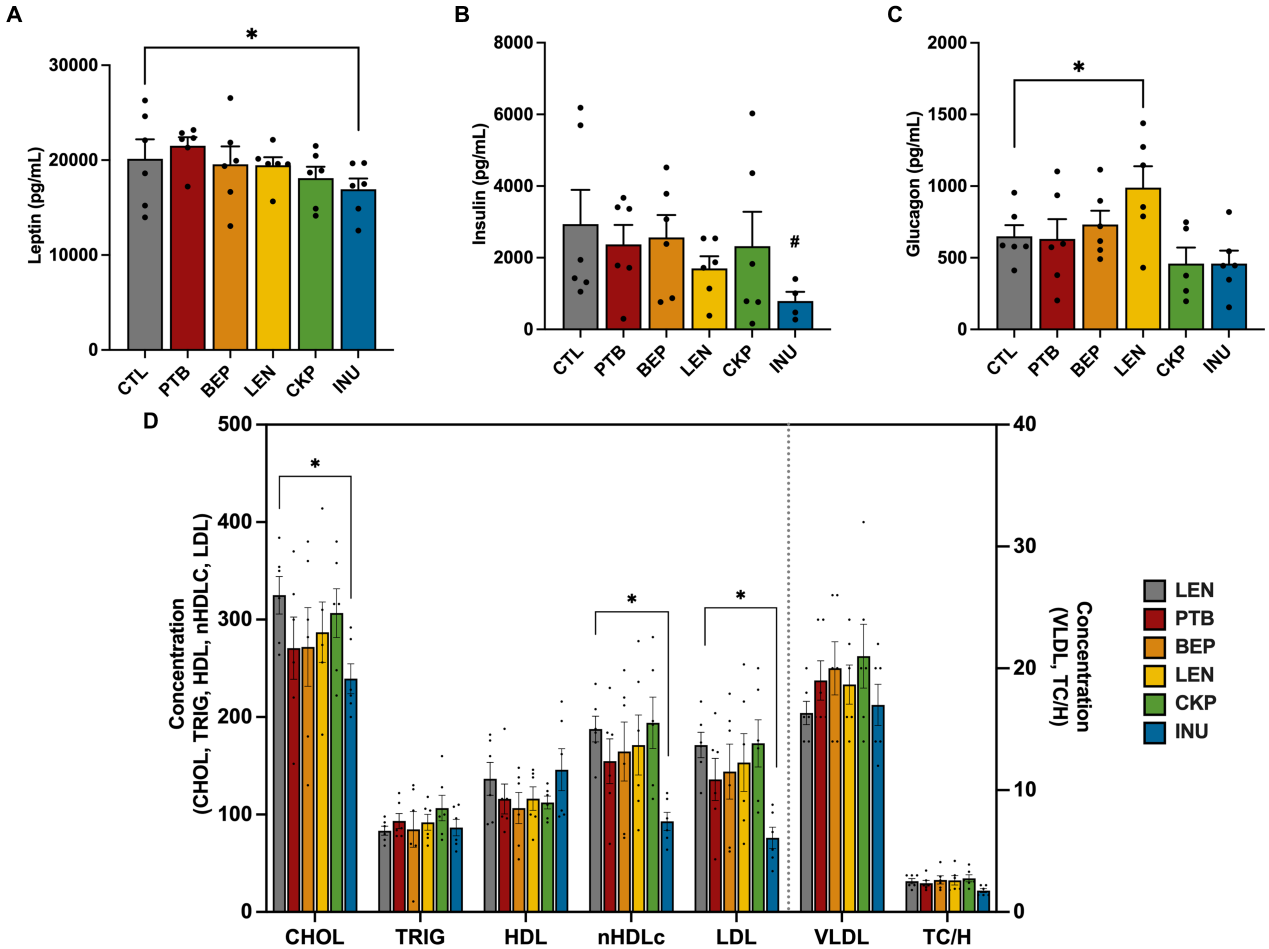
Influence of RS and inulin supplementation on the regulation of endocrine response and lipid profiles in serum. **(A)** Leptin levels, **(B)** Insulin levels, **(C)** Glucagon levels, and **(D)** Lipid profiles. CTL, control group; PTB, pinto beans; BEP, black-eyed-peas; LEN, lentils; CKP, chickpeas; INU, inulin. Data includes both genders; pairwise comparisons adjusted for sex. *p < 0.05, ^#^*p* < 0.10.

The changes in serum concentrations of various markers involved in appetite regulation and energy expenditure, following consumption of an RS-supplemented diet, are presented in Figures 4A–C. The overall impact of RS-supplemented diet groups on serum hormonal and lipid profiles was ascertained through ANCOVA after adjusting for sex, the results of which are presented in Supplementary Table S3. Despite no significant main effect (*p* = 0.064) of diet groups on leptin levels, a decreasing trend was observed in all RS groups except for PTB, with significant reductions (*p* = 0.038) observed in the INU group compared to the CTL group (Figure 4A). Leptin concentration differed significantly (*p* < 0.001) across sex with females exhibiting a significant reduction (*p* < 0.05) in leptin levels, particularly observed in the CKP and INU groups (Supplementary Figure S4A). Insulin levels were not significantly affected by diet groups overall (*p* = 0.445); however, the INU group exhibited a mild suppression of insulin levels (*p* = 0.085) (Figure 4B). On the other hand, glucagon levels were significantly influenced by diet group (*p* = 0.024), with the LEN group showing higher levels (*p* = 0.036) compared to the CTL group overall (Figure 4C). Besides, LEN group also had consistently higher glucagon levels than CTL in both sexes (Supplementary Figure S4C). Collectively, the endocrinal responses of RS in regulating glucose and appetite metabolism found more pronounced in females than males, which were majorly associated with LEN, CKP and INU groups.

The impact of RS intervention in regulating lipid metabolism was assessed via quantitative determination of lipids and lipoproteins in mice serum as depicted in Figure 4D. Although the diet groups have a marginal influence on cholesterol levels (*p* = 0.097), only INU group exerted significant reductions (*p* < 0.05) in the cholesterol levels overall. The diet groups did not induce significant changes in the levels of triglycerides, HDL, VLDL, and TC/H ratio. However, notable reductions were observed in nHDLc (*p* = 0.041) and LDL (*p* = 0.035) levels, particularly in the INU group. Such lowering effect of INU was also consistent in both sexes (Supplementary Figures S4D,E). Additionally, we also analyzed serum levels of liver function enzymes (ALT and AST) and glucose in combined and individual sexes (Supplementary Figures S4F–H). AST levels were significantly impact by diet groups (*p* = 0.031) while ALT levels were mildly influenced (*p* = 0.087). INU group exhibited significant improvements (*p* < 0.05) in liver function, as evidenced by reduced levels of ALT and AST in the combined sex group mice (Supplementary Figure S4F) and in females’ mice (Supplementary Figure S4G) specifically. Surprisingly, PTB significantly (*p* < 0.05) enhanced levels of ALT and AST compared to CTL in the male mice (Supplementary Figure S4H). Although there was no significant overall impact of diet group (*p* = 0.203) on serum glucose levels, the CKP group was found to significantly lower (*p* < 0.05) glucose levels compared to the CTL group (Supplementary Figure S4F). Altogether, INU group exhibited prominent changes in lipid metabolism and liver function enzymes relative to control group.

### 3.4 Microbiome-metabolome interactions with neurobehavioral, neuroinflammatory, endocrinological, and metabolic responses

**Figure 5:**
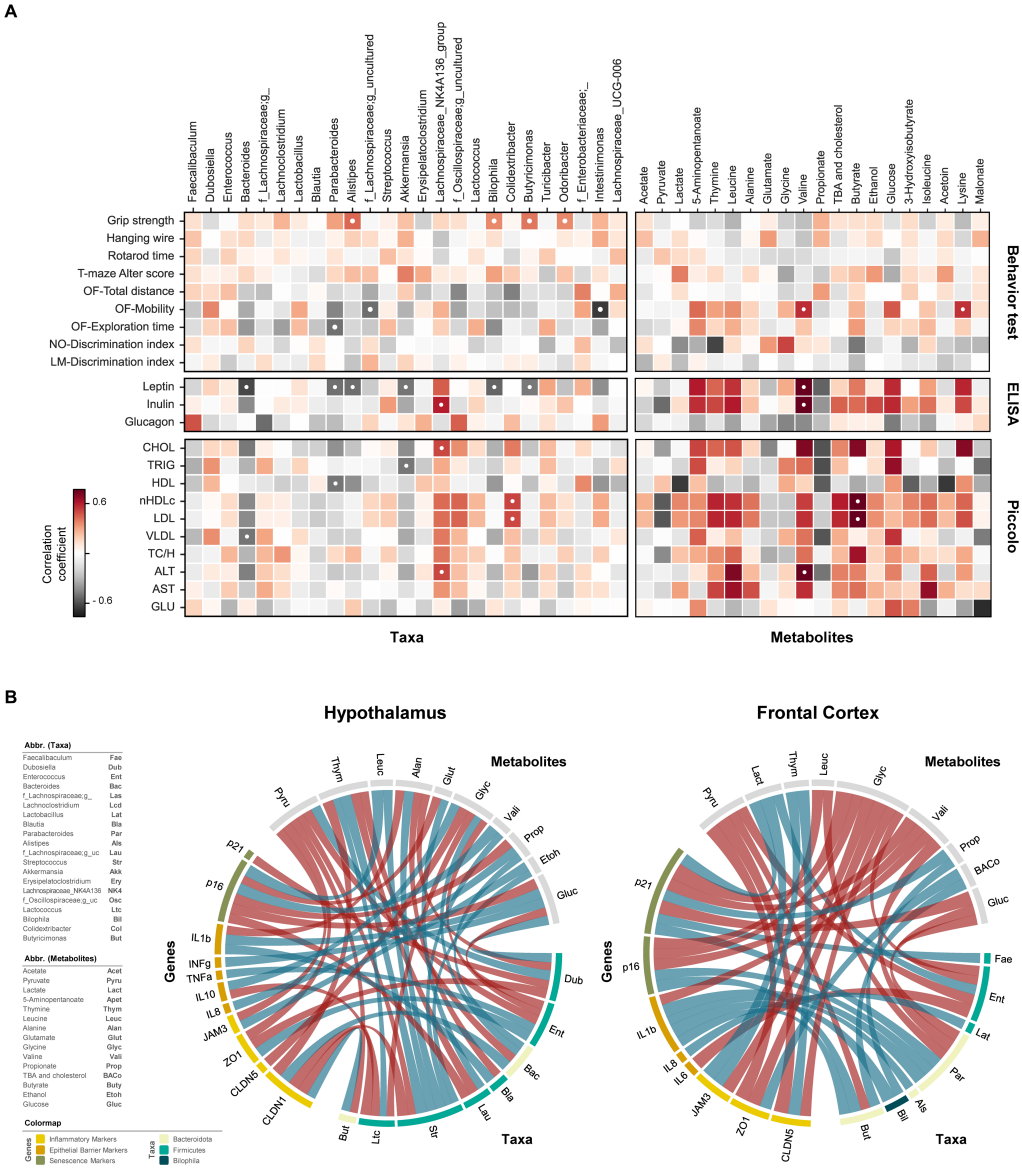
Interplay of gut microbiome and metabolome in regulating cognitive functions, neuroinflammation, endocrinolgical, and hepatic health markers. **(A)** Heatmaps showing correlational relationships between neurocognitive features (behavior test), serum markers (ELISA measurements), and blood lipid profiles (Piccolo measurements) with respective gut bacterial genera and metabolites. **(B)** Circos plots depicting relationships between gut microbiome genera and microbial metabolites with host genes in the hypothalamus and frontal cortex. Negative correlations are represented by red lines; positive correlations are denoted by blue lines. ● *p* < 0.05, adjusted with Benjamini-Hochberg correction.

The systemic effects of RS-driven modulation of the gut microbiome and metabolome on the neurobehavioral, endocrinological, and brain gene markers was assessed using spearman’s correlational analyses using top 25 genera and top 20 metabolites as summarized using heatmap (Figure 5A) and circos plot (Figure 5B). Among the neurobehavioral parameters, grip strength was positively correlated with *Alistipes, Bilophila, Butyricimonas,* and *Odoribacter*. Mobility exhibited a strong negative correlation with *Intestinimonas* and *f_Lachnospiraceae*, and a strong positive correlation with lysine and valine. Longer exploration times in an open field was negatively related with *Parabacteroides*, while a non-significant positive association of same was also observed with butyrate and 5-aminopentanoate. None of the microbiome signatures gave significant correlation with T-maze alteration score, and discrimination indices of novel object and location memory tests, however, some tendencies were noted for metabolites. For instance, T-maze alteration score was positively correlated with lactate, ethanol, and acetoin, while an inverse association existed with glycine and glucose. Metabolites *viz.*, thymine, butyrate, lactate, and 5-aminopentanote found negatively associated with novel object discrimination, while glutamate and glycine showed positive association with same.

Among endocrinological markers, leptin was inversely associated with *Bacteroides*, *Parabacteroides, Alistipes, Bilophila, Butyricimonas,* and *Akkermansia*, whereas insulin exhibited positive association with *Lachnospiraceae_NK4A136 group*. Valine correlated positively with both leptin and insulin, while propionate displayed a non-significant negative association with these markers. Serum lipid profiles demonstrated intricate relationship with microbiome and metabolome. Evidently, *Bacteroides* and *Lachnospiraceae_NK4A136 group* had opposite effects on lipid markers. *Bacteroides* showed negative associations, while *Lachnospiraceae_NK4A136* displayed positive associations with CHOL, nHDLc, LDL, VLDL, and ALT profiles. Nevertheless, the significance of these associations was only evident for CHOL and ALT in the case of *Lachnospiraceae_NK4A136*, and for VLDL in the case of *Bacteroides.* Positive association of nHDLc and LDL with *Colidextribacter* and butyrate was observed. Despite non-significant, nHDLc and LDL levels were also positively linked with thymine, leucine, total bile acids (TBA) and cholesterol but negatively with pyruvate. Triglycerides were negatively correlated with *Akkermansia*. TBA and cholesterol showed a weak positive association, though not statistically significant, with various cholesterol and liver enzyme markers, except for HDL, which exhibited a slight negative association trend. Propionate also exhibited a non-significant negative trend with various lipid profiles and liver markers. Valine was strongly related to higher presence of liver enzymes (ALT and AST).

The direct and indirect relationship between gene markers of hypothalamus and FC, gut microbiome and fecal metabolites were evaluated using circus plot (Figure 5B). In the hypothalamus, thymine was the only metabolite negatively associated with CLDN1 expression. Besides, *Bacteroides* and *Butyricimonas* had a positive effect on its expression, while *Streptococcus* and *Lactococcu*s had a negative effect. Furthermore, the abundance of thymine was also positively and negatively associated *Streptococcus* and *Bacteroides*, respectively. Glycine exhibited positive and negative correlations with IL10 and CLDN5, respectively. *Lactococcus* had direct inverse relationship on IL10 and glycine. Abundance of *Bacteroide*s was directly correlated with glycine levels, suggesting the indirect effect of this genus on IL10 expression. The direct positive interaction between *Dubosiella* and pyruvate, inversely associated with ZO1 expression. However, the expression of ZO1 and TNF⍺ correlated positively with *Enterococcus*, which itself had a negative association with pyruvate, glucose, and glycine abundance. The inverse relationship between pyruvate and TJPs (JAM3 and ZO1) could be indirectly attributed to its opposite association with the abundance of *Streptococcus*, *Enterococcus*, and *Butyricimonas*. IFNɣ exhibited only direct association with glucose, while IL1β expression straightly influenced by both glucose and *Blautia*. Propionate, whose abundance is directly linked with *Bacteroides*, positively associated with IL8, and inversely associated with p16 expression. Furthermore, p16 expression was positively related to valine and leucine, which are directly correlated with *Streptococcus*, suggesting its potential role in accelerating senescence. *Dubosiella* also had an inverse association with p21, another senescence-related marker.

In the FC, all three epithelial barrier markers (CLDN5, ZO1, and JAM3) were negatively correlated with glycine. Additionally, CLDN5 and ZO1 exhibited a negative association with pyruvate, while CLDN5 and JAM3 were positively related to thymine. TBA and cholesterol were positively related to ZO1 expression and *Butyricimona*s. Among inflammatory markers, the expression of IL6 and IL8 were negatively and positively associated with *Enterococcus* and *Butyricimonas*, respectively. Apart from valine metabolite, the expression of IL1β was directly associated with *Bilophila, Alistipes*, *Butyricimonas,* and *Parabacteroides*. The latter two also had positive relationship with p16, while a negative association of p16 with glycine, valine and leucine was observed. Positive correlation of p21 revealed with *Parabacteroides, Faecalibaculum*, lactate and propionate, while negative correlation found with glucose, valine and glycine. Notably, propionate abundance in this case was associated with *Biophilia*, suggesting its consequential involvement in promoting senescence. Besides, *Enterococcus* might be indirectly involved with senescence and barrier integrity in FC owing to its relationship with glycine, lactate, and pyruvate. The detailed associations of brain genes with gut microbiome and metabolite fingerprints were also depicted via heatmap (Supplementary Figure S5). Taken together, these findings suggest that the gut microbiome, through the gut-brain axis, could have significant impacts on neurobehavioral, endocrinological, and gene expression profiles in brain tissues. This effect may be explained via the translocation of gut derived metabolites to brain and/or direct interactions with enteroendocrine cells, eventually influencing the neurocognitive attributes.

## 4 Discussion

Aging-associated neurocognitive decline is a significant contributor of poor lifestyle and rising health care costs among the elderly. Increasing evidence suggests that improving the lifestyle factors especially diet can either prevent or ameliorate the symptoms of dementia in old age (28). Diet plays a critical role in modulating the gut microbiome, which, in turn, can impact neural functions through various mechanisms within the gut-brain axis. For instance, high-fat and fiber-deprived dietary patterns can induce deficits in learning, memory and executive functions which are associated with disruptions in neurochemical, endocrinological and inflammatory tone mediated by obesity-associated microbiota (7). On the contrary, incorporation of dietary fibers manipulates the gut microbiome towards a homeostatic state with enhanced proportion of beneficial members and metabolites thereby improving neurocognition (21). Our findings suggest that the supplementation of dietary pulses-derived resistant starches can prevent/ameliorate the western-style HFD-induced neurobehavioral dysfunctions in this “humanized” mouse model of aging, i.e., older mice carrying the microbiome of human older adults. Further, improvements in neurocognition differ among the RS source and are associated with gut microbiome-metabolome remodeling, which, in turn, induce changes in neuroinflammation, cellular senescence, and endocrinological signaling.

Given the strong associations between gut dysbiosis, obesity, and cognitive decline, we observe attenuation of cognitive impairments induced by HFD-fed aged mice by RS supplementation, more prominently by LEN group. LEN group demonstrated superior cognitive and explorative functions in T-maze and open-field tests, respectively. Evidence of HFD-associated microbiota in reducing exploratory behavior during open-field test has also been reported previously (29). Earlier studies have reported ameliorative effects of β-glucan supplementation on spatial memory impairments in diet-induced obese mice by enhancing neurobehavioral performance in novel object, location memory and Y-maze tests (21, 30). Moreover, whole seed extracts of Adzuki beans have been reported to confer neuroprotective effects by enhancing spatial and recognition abilities in HFD-fed mice as evaluated using T-maze and novel-object tests (31). While we observe a positive discrimination index in object location, the negative discrimination in novel object across all groups could potentially be attributed to the mice’s preference for familiar object, which might be governed by the size, shape, odor, texture or brightness of the object (32). Nevertheless, we observe positive associations of butyrate, propionate and/or lactate (an important contributor of SCFA production) with cognitive and exploratory behaviors. Microbiota-SCFA-brain axis has repeatedly been linked with improvements in behavioral outcomes associated with dietary fiber intake (8, 33). Recently, butyrate, but not propionate, was shown to effectively reverse the neurocognitive deficits and synaptic plasticity in the offspring of dams on fiber-deprived diet, putatively via inhibition of histone deacetylase (HDAC-4) expression (34). Our preceding studies also demonstrated significantly higher levels of butyrate in the LEN group compared to the other groups as used in this study (17). These findings suggest a potential mechanism underlying the superior neurobehavioral features observed in mice fed with RS from lentils.

Another potential mechanism of bidirectional crosstalk between gut-brain axis involves immune regulation. Gut dysbiosis could trigger a pro-inflammatory cascade leading to neuroinflammation, which is a central pathophysiological hallmark of neurodegenerative diseases (30). Neuroinflammatory response is closely associated with impaired blood–brain-barrier (BBB) permeability (35). Accordingly, we herein evaluate the expression of TJPs in different regions of brain involved in cognitive functions, wherein we observe sexual dimorphism in relative expression of proteins, especially for CLDN1, CLDN5 and ZO1. RS-mediated expression of TJPs seems to benefit females more than males. Earlier studies have demonstrated that xylooligosaccharides supplementation to a surgery-induced cognitive dysfunction in a APP/PS1 male mice upregulates ZO1 and occludin in the hippocampal region of brain (36). Moreover, we also observe suppression of pro-inflammatory mediators *viz.*, IFNɣ, IL6, IL8, TNFα and IL1β in either one or both sexes for different RS groups, indicating the modulatory role of RS in ameliorating the BBB disruption and neuroinflammation. The role of gut-immune axis has been implicated in various neurodegenerative and psychiatric disorders. Gut dysbiosis can adversely affect the immune system maturation, leading to a shift towards a pro-inflammatory phenotype whereby released cytokines and chemokines bind to endothelial cells of brain via disrupted BBB, and cause elevated immune responses (37). Microbiome-stimulated production of TNFα and IL1β has previously been reported to impair hippocampal-dependent memories in obese mice (30). The proinflammatory cytokine IL6 usually increases with age and has been associated with cognitive impairments (38). Elevated serum levels of IL8 have been linked to poorer cognition and motor function in elderly individuals (39). This could potentially explain LEN-induced downregulation of IL8 in the brain tissues and associated improved neurobehavior performance in both sexes. However, the effect of IL8 on cognitive functions remains somewhat complicated, as it can also act as neuroprotective at optimal concentrations (40). Inulin has been shown to reverse stress-induced peripheral and neuro-inflammation via gut microbiome modulation in middle-aged mice (41). The precise mechanisms of sex dimorphism in immunological attributes are unclear; however, the sex differences in gut microbiome composition and immune responses as well as their relationship with sex hormones have been acknowledged (42). Microglia, the key regulators of inflammation in the brain, exhibit sex-specific functions which may uniquely affect the progression of neurodegenerative diseases among sexes (43). Our previous studies also demonstrated that the systemic effects of prebiotics on gut and metabolic health differ between males and females (18, 44).

Microbiome-metabolome correlational networks with immune mediators provide deeper insights into complex crosstalk with immune system. Intriguingly, we observe association of thymine in reducing the expression of claudins in the hypothalamus. Such an association has been demonstrated in Alzheimer’s mouse models, whereby oral administration of *Faecalibacterium prausnitzii* reduced thymine levels in the hippocampus along with amelioration of cognitive impairments (45). Although we did not measure thymine levels in the brain, its abundance had been reported in the brain of Alzheimer’s disease (AD) patients and might be associated with elevation of oxidative stress (46). The positive association of glycine with IL10 also aligns well with a previous study (47), wherein glycine exhibited anti-inflammatory effects by reducing colonic expression of IL1β and promoting IL10 cytokines in a colitis mouse model. We also observed indirect association of *Bacteroides* in increasing IL10 levels in hypothalamus via glycine upregulation. Relatively higher expression of IL10 in hypothalamus for CKP and INU groups could be partly attributed to higher abundance *Bacteroides* in these groups as per our earlier study (18). The microbiome, specifically bacteria including *Bacteroides, Bifidobacterium, Clostridium*, and *Lactobacillus*, can produce glycine by breaking down bile acids using bile salt hydrolase (BSH). Changes in the levels of these bacteria have been observed in individuals with AD, indicating a possible link between the microbiome, bile acids, and brain signaling in influencing cognitive health (48). Alternatively, the polysaccharide A of *Bacteroides fragilis* can directly induce differentiation of naïve CD4+ T cells to IL-10 producing FoxP3 + Treg cells, thereby conferring protection against CNS demyelinating diseases (49).

The gut microbiome plays a crucial role in the intricate relationship between inflammaging, cellular senescence, and aging. Dietary interventions aimed at remodeling the microbiome have the potential to mitigate age-related complications in the gut (50). Cyclin-dependent kinase inhibitors p16 and p21 serve as valuable markers for cellular senescence, with their levels rising in tandem with the aging process (51). Our data show significant lower expression of senescent markers in aged mice after dietary RS intervention compared to control. Recent studies have reported benign effects of dietary genistein in reducing the mRNA expression levels of p16 and p21 in older mice, which were associated with the effect of SCFAs (butyrate and propionate) in mitigating TNF⍺-induced intestinal damage (52). Our findings also demonstrate inverse relationship of propionate with p16 in the hypothalamus wherein the treatment groups exhibit lowest p16 expression compared to control. Additionally, we also find either positive or negative associations of fecal branched-chain amino acids (BCAAs) i.e., valine and leucine, with senescence markers in the brain. BCAAs can act as signaling molecules and perform a variety of cell functions, but their exact mechanisms in regulating the aging process are not fully elucidated (53). For instance, leucine supplementation, in one study, displayed neuroprotective effects by reducing release of senescence-associated secretory phenotype (SASP) from senescent astrocytes (54). On the other end, overaccumulation of leucine may induce cancer cell proliferation, inflammation, and neurodegeneration (55, 56). Apart from ingested diet, gut microbial biosynthesis is also implicated in production of BCAAs. Our correlational analyses reveal indirect association of *Streptococcus* in increasing p16 in the hypothalamus via promoting fecal valine and leucine levels. We have previously shown reduced relative abundance of *Streptococcus* and BCAAs after RS intervention (17, 18). Similar effects in ameliorating the gut dysbiosis and reducing peripheral BCAAs by dietary berberine (a phytochemical) in HFD-fed mice have been reported in other studies (57). Recent studies have also highlighted higher abundance of *Streptococcus* and lower abundance of *Bacteroides* in patients with cognitive impairment compared to healthy controls (58). In summary, these data indicate that the RS exerts beneficial effects on gut-brain signaling in these aged mice via remodeling of the gut microbiome-metabolome signatures, resulting in the regulation of BBB integrity, reduction of pro-inflammatory mediators, and cellular senescence markers.

We observe that the inclusion of prebiotics (RS and inulin) in the high-fat diet improves the hormones associated with energy balance, glucose, and appetite metabolism. Moreover, these endocrinological signals are also tightly regulated by the interactive effects of the gut microbiome and metabolome, which are eventually sensed by brain, thus facilitating appetite and energy balance of the host. Specifically, we observe a decrease in serum concentrations of leptin, particularly in the CKP and INU groups. Insulin reduction is only appreciable for INU among all treatment groups. Leptin, a cytokine-like hormone, is majorly synthesized by adipocytes which is involved in satiety regulation and energy expenditure. HFD-induced hyperleptinemia contribute to the burden of obesity and insulin resistance by impairing the leptin signaling (59). Previous studies have exhibited reduction in leptin levels in subjects consuming HFD after resistant starches (60), and dietary fibers intake (61). Inulin has been reported to restore impaired leptin gene signaling especially via gut-derived SCFAs’ activation of AMPK pathway leading to enhanced fatty acid oxidation with concomitant inhibition of hepatic gluconeogenesis and cholesterol synthesis (62). Dietary supplementation of SCFA has been demonstrated to reduce leptin levels in diet-induced obese mice via regulation of G-protein coupled receptors (63). In line with this, we also observe negative correlation of propionate and propionate-producers *viz.*, *Bacteroides* and *Akkermansia* with leptin levels. Interestingly, Gunstad et al. (64) found a positive association of elevated leptin levels and age-related cognitive decline in older adults, further implying the beneficial effects of prebiotics in improving neuronal function via gut hormones signaling. Glucagon, a hormone secreted by the alpha cells of the pancreas, functions to stimulate the liver and promote glucose synthesis, while also counteracting the actions of insulin. Although high glucagon levels are associated with insulin resistance (65), given the relatively higher glucagon levels in LEN group compared to CTL, we did not observed insulin resistance in our previous study (18). Nevertheless, this suggests the interactive effects of other metabolic markers in maintaining the glucose homeostasis. Besides, the dynamic and intricate balance between insulin and glucagon may be subject to influence from several factors including blood glucose levels, dietary fiber type and its fermentation, presence of free sugars in the fiber, and the rate of nutrient absorption.

We observe beneficial modulation of serum lipid profiles and liver function enzymes by prebiotics; however, INU group exerts superior regulation of these markers compared to RS groups. The pronounced effects of INU on lipid metabolism could be attributed to its well-purified form, which was discreetly utilized by specific bacterial members, including *Bacteroides*. This utilization resulted in the production of propionate as a SCFA, whose positive effects on inhibiting cholesterol synthesis, owing to its involvement in hepatic gluconeogenesis, are well-recognized (66). The significant downregulation of serum concentrations of CHOL, nHDLc and LDL by INU group is in concert with previous studies (67), wherein inulin treatment was shown to alleviate hyperglycemic effects in diet-induced diabetic mice. Additionally, such effects are attributed to the promotion of *Bacteroides* genus, a correlation that was also observed in our current study. The ameliorative effects of inulin on lipid metabolism could be partly attributed to reduction in HFD-induced liver damage. The bloodstream levels of liver enzymes (ALT and AST) are usually increased if liver is damaged (68). Reductions in level of liver enzymes by INU group are consistent with earlier findings (69). Despite non-significant, RS groups, especially in females, also seem to exhibit decreased tendency in above markers, thereby suggesting improvements in lipid metabolism relative to control. Previous studies have reported improvements in the hyperlipidemia after 6-weeks of intragastric administration with autoclaved and unautoclaved lentil starch to HFD-fed mice, with the former one exhibiting more pronounced effects than later (20). The milder effects observed in current study could be attributed to the dosage, mode of administration and type of resistant starch. We also observe positive association of BCAAs with liver enzymes. Gut microbiome also regulates BCAAs synthesis via gut-liver axis, wherein low microbial diversity associates with increased levels of BCAAs which, in turn, promotes the progression of metabolic and hepatic disorders (70). Noteworthily, we have recently demonstrated a positive association of BCAAs with liver weight and an inverse association with RS supplementation (17). Besides, we observe lower serum glucose levels in CKP group, which correlates well with its positive effects in reducing post-prandial glycemia as also reported previously (18). Improvements observed in these metabolic conditions are also relevant from the viewpoint of gut-brain axis, particularly given that several associative studies have predicted metabolic syndrome and/or obesity as risk factors for the progression of neurocognitive decline and dementia (7, 71, 72).

Taken together, we investigate how prebiotics’ fermentation in the gut may initiate a series of interactions, including immunological, endocrinological, and metabolic signaling pathways, ultimately leading to enhanced brain health. Our findings demonstrate resistant starches derived from various dietary beans and pulses can attenuate neurocognitive impairments in older mice carrying human gut microbiome. However, the extent of this attenuation varies, as not all resistant starches (RS) equally generate long-term improvements in cognitive function. For instance, RS derived from lentils demonstrates visible improvements in neurobehavioral performance, while no noticeable effects were detected for the RS derived from black-eyed peas in the current study. This could be potentially linked to the RS-mediated unique orchestration of gut microbiome and metabolome and their subsequent impacts on gut-brain axis. The mechanisms associated with such improvements in neurocognitive features may include modulation of neuroinflammatory tone by suppressing proinflammatory cytokines (IFNɣ and IL6) and chemokines (IL8); restricting cellular senescence (p16 and p21); attenuating heightened levels of gut hormones (leptin and insulin); and improving lipid metabolism (e.g., reducing cholesterol levels) by restoring liver function. These beneficial outcomes can be attributed to the fostering of beneficial genera (e.g., *Bacteroides*) and suppression of potentially determinant genera (e.g., *Streptococcus*) together with enhancement and diminution of SCFAs (butyrate) and BCAAs (leucine), respectively. Overall, these findings demonstrate the neuroprotective potential of specific RSs by elucidating potential and intricate mechanisms of gut microbiome-metabolome remodeling, offering promising avenues for enhancing brain/neurocognitive health in aging populations. These data should inspire and propel future studies to deeply investigate specific neurotransmitters and synaptic plasticity-associated proteins involved in memory regulation and shed light on mechanisms underlying the RS- or fiber-mediated modulation of neurocognitive health via the diet-microbiome-brain axis.

## Data availability statement

All datasets generated for this study are included in the article/Supplementary material. All the raw sequencing datasets have been submitted to the NCBI Sequence Read Archive (SRA) public repository database under SRA BioProject number PRJNA902407.

## Ethics statement

The animal study was approved by Florida State University Institutional Animal Care and Use Committee (PROTO202100008). The study was conducted in accordance with the local legislation and institutional requirements.

## Author contributions

SK: Data curation, Formal analysis, Investigation, Methodology, Software, Visualization, Writing – original draft. GP: Data curation, Formal analysis, Methodology, Software, Visualization, Writing – review & editing. NH: Formal analysis, Methodology, Writing – review & editing. KM: Formal analysis, Methodology, Writing – review & editing. BW: Formal analysis, Methodology, Writing – review & editing. RN: Conceptualization, Funding acquisition, Investigation, Project administration, Supervision, Writing – review & editing.
