# Supplementary material for: Resistant starches from dietary pulses improve neurocognitive health via gut-microbiome-brain axis in aged mice
Source: Front Nutr. 2024 Jan 24;11:1322201. doi: 10.3389/fnut.2024.1322201 (PMC10864001; doi:10.3389/fnut.2024.1322201)

Supplementary Table S1. List of primers used in current study for qPCR analysis

Gene name	Forward primer	Reverse primer	Reference#
18S	AGAAACGGCTACCACATCCA	CCCTCCAATGGATCCTCGTT	(1)
CLDN1	GGCTTCTCTGGGATGGATCG	CTTTGCGAAACGCAGGACAT	(1)
CLDN5	GTTAAGGCACGGGTAGCACT	TACTTCTGTGACACCGGCAC	(1)
ZO1	AAGAAAAAGAATGCACAGAGTTGTT	GAAATCGTGCTGATGTGCCA	(1)
OCCL	CTGACTATGCGGAAAGAGTTGAC	CTGACTATGCGGAAAGAGTTGAC	(1)
JAM3	GCTGTGAGGTCGTTGCTCTA	AGTGGCACATCATTGCGGTA	(1)
IL1β	GAAATGCCACCTTTTGACAGTG	TGGATGCTCTCATCAGGACAG	(2)
IL6	GTCCTTCCTACCCCAATTTCCA	CGCACTAGGTTTGCCGAGTA	(1)
IL8	ACTCAAGAATGGTCGCGAGG	GTGCCATCAGAGCAGTCTGT	(1)
IL10	TGGGTTGCCAAGCCTTATCG	TTCAGCTTCTCACCCAGGGA	(1)
IFNɣ	CAGCAACAGCAAGGCGAAA	CTGGACCTGTGGGTTGTTGAC	(1)
TNF-α	GATCGGTCCCCAAAGGGATG	TTTGCTACGACGTGGGCTAC	(1)
p16	CCCAACGCCCCGAACT	GCAGAAGAGCTGCTACGTGAA	(3)
p21	GTCAGGCTGGTCTGCCTCCG	CGGTCCCGTGGACAGTGAGCAG	(3)

#(1) Miranda-Ribera A, Ennamorati M, Serena G, Cetinbas M, Lan J, Sadreyev RI, Jain N, Fasano A, Fiorentino M. Exploiting the zonulin mouse model to establish the role of primary impaired gut barrier function on microbiota composition and immune profiles. *Front Immunol*. (2019) 10:2233.

(2) Minato KI, Ohara A, Mizuno M. A proinflammatory effect of the β-glucan from *Pleurotus cornucopiae* mushroom on macrophage action. *Mediators Inflamm*. (2017) 22:2017.

(3) Saccon, T. D., Nagpal, R., Yadav, H., Cavalcante, M. B., Nunes, A. D. D. C., Schneider, A., ... & Masternak, M. M. (2021). Senolytic combination of dasatinib and quercetin alleviates intestinal senescence and inflammation and modulates the gut microbiome in aged mice. *The Journals of Gerontology: Series A*, *76*(11), 1895-1905.

Supplementary Table S2A: Linear regression analysis for novel object and location memory test.

Predictors	Coefficients				Model Summary			
	B	Standard error	t-statistic	Significance	R square	Adjusted R square	F	Significance
**Novel Object Test**								
Intercept	5.072	.766	6.621	<.001	0.478	0.319	2.998	0.006
Time-familiar	-.599	1.087	-.551	.585				
Diet group-PTB	.596	1.099	.542	.591				
Diet group-BEP	-1.119	1.156	-.969	.339				
Diet group-LEN	-2.040	1.101	-1.853	.072				
Diet group-CKP	-2.102	1.260	-1.668	.104				
Diet group-INU	.086	1.025	.084	.933				
Interaction-PTB*time-familiar	2.097	1.327	1.581	.123				
Interaction-BEP*time-familiar	4.982	1.579	3.156	.003				
Interaction-LEN*time-familiar	-.242	1.220	-.198	.844				
Interaction-CKP*time- familiar	.423	1.191	.355	.725				
Interaction-INU*time-familiar	.614	1.614	.381	.706				
**Location Memory Test**								
Intercept	6.640	1.489	4.459	<.001	0.358	0.151	1.726	0.109
Time-familiar	-.184	2.160	-.085	.933				
Diet group-PTB	3.392	2.389	1.420	.165				
Diet group-BEP	-.770	2.289	-.336	.739				
Diet group-LEN	-1.284	2.457	-.523	.605				
Diet group-CKP	.166	2.109	.079	.938				
Diet group-INU	1.089	2.640	.413	.683				
Interaction-PTB*time-familiar	-1.146	2.933	-.391	.698				

Interaction-BEP*time-familiar	2.094	3.451	.607	.548				
Interaction-LEN*time-familiar	3.254	2.962	1.099	.280				
Interaction-CKP*time- familiar	-1.606	3.669	-.438	.664				
Interaction-INU*time-familiar	1.538	2.960	.520	.607				

Supplementary Table S2B: ANCOVA results for novel object and location memory test.

**Variables**	**F**	**Significance**	**Partial Eta squared**
**Novel Object Test**			
Corrected Model	1.899	.104	.217
Intercept	17.643	<.001	.301
Time familiar object (covariate)	5.813	.020	.124
Diet group	1.270	.295	.134
**Location Memory Test**			
Corrected Model	1.853	.114	.222
Intercept	5.416	.025	.122
Total original object location (covariate)	8.849	.005	.185
Diet group	.663	.654	.078

Supplementary Table S3: ANCOVA results for serum hormonal and lipid profiles, using sex as a covariate.

Dependent variable	Independent variable/ covariate	F	Significance
**Serum hormonal profiles**			
Leptin	Diet group	2.370	0.064
	Sex	26.694	<0.001
Insulin	Diet group	0.985	0.445
	Sex	2.040	0.165
Glucagon	Diet group	3.090	0.024
	Sex	3.011	0.094
**Serum lipid profiles**			
CHOL	Diet group	2.080	0.097
	Sex	27.731	<0.001
TRIG	Diet group	0.709	0.622
	Sex	6.068	0.020
HDL	Diet group	1.809	0.142
	Sex	25.360	<0.001
LDL	Diet group	2.793	0.035
	Sex	4.251	0.048
VLDL	Diet group	1.307	0.288
	Sex	17.830	<0.001
nHDLc	Diet group	2.690	0.041
	Sex	5.122	0.031
TC/H	Diet group	2.222	0.079
	Sex	0.358	0.554
ALT	Diet group	2.154	0.087
	Sex	17.417	<0.001
AST	Diet group	2.881	0.031
	Sex	2.567	0.120
GLU	Diet group	1.559	0.203
	Sex	0.500	0.485

Supplementary Figure S1. **(A)** Total distance travelled, and **(B)** Mobility in an open field test for different RS and inulin groups. Neurobehavioral outcomes for different groups presented sex-wise: **(C)** Grip strength, **(D)** Rotarod test, **(E)** Hanging wire test, **(F)** T-maze spontaneous test, **(G)** Open field test, **(H)** Novel object test, and **(I)** Location memory test. #p<0.10

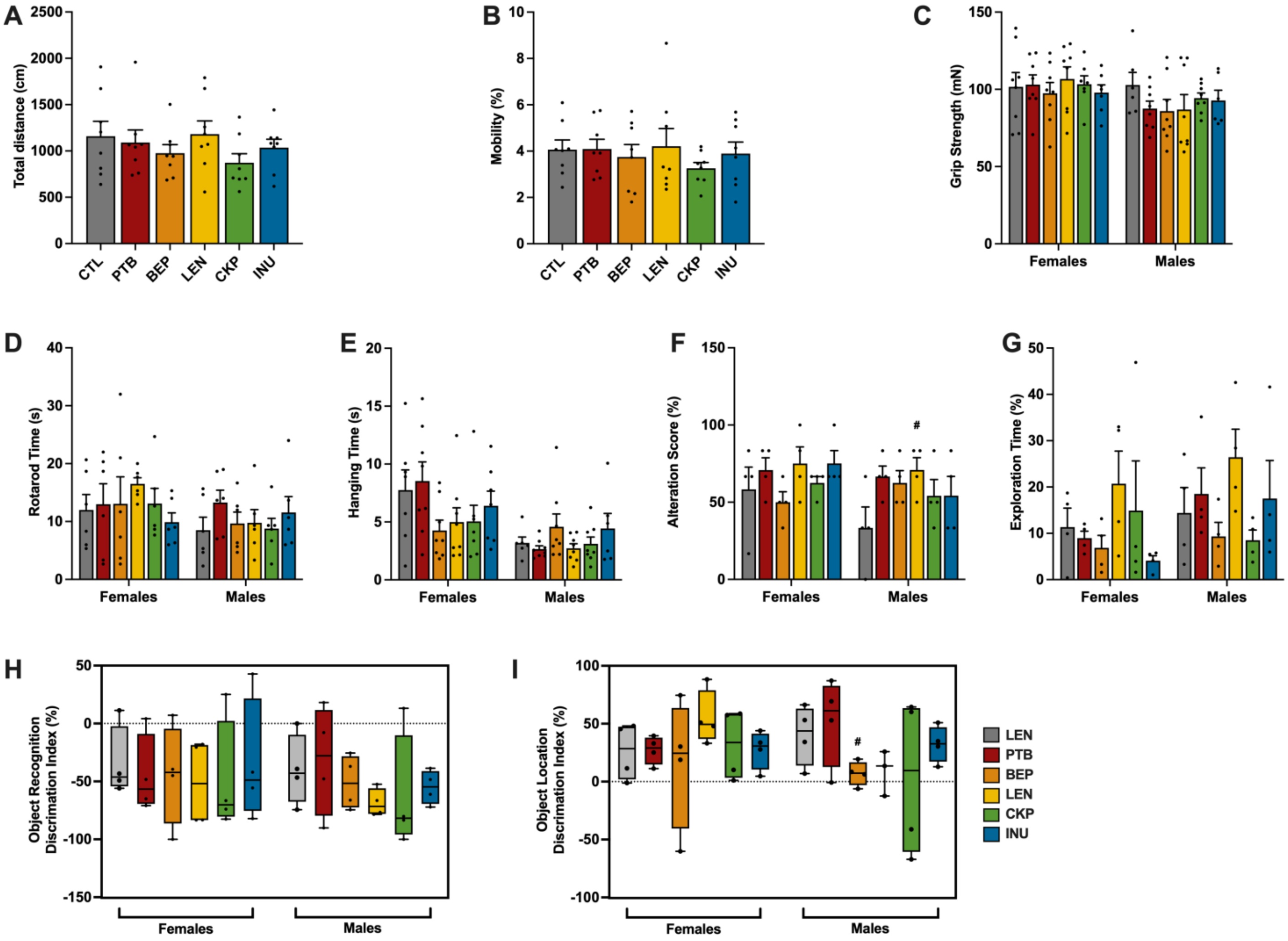

Supplementary Figure S2. Heatmap illustrating the influence of treatment groups and brain regions on the expression of tight junction proteins, inflammatory and senescent markers. Statistical significance for each gene was determined through a two-way ANOVA after rank transformation of fold change data. •$p<0.05$; ••$p<0.01$; •••$p<0.001$

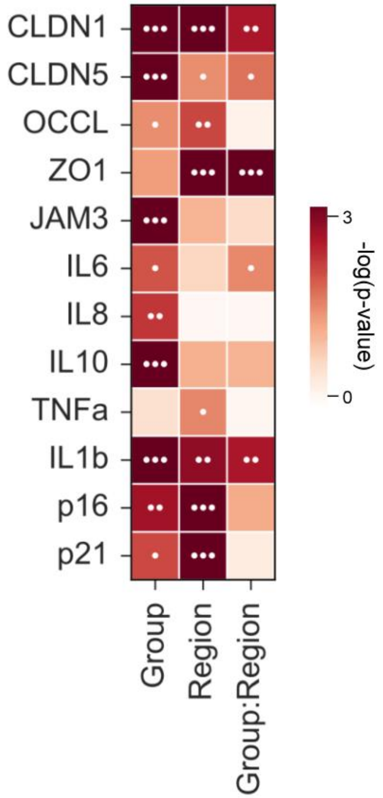

Supplementary Figure S3. Sex-specific changes in the expression of tight junction protein in (the **A)** Hypothalamus, **(B)** Frontal cortex; inflammatory markers in the **(C)** Hypothalamus, **(D)** Frontal cortex; and cellular senescence markers in the **(E)** Hypothalamus, **(F)** Frontal cortex, **(G)** Colon, and **(H)** Ileum. *p<0.05.

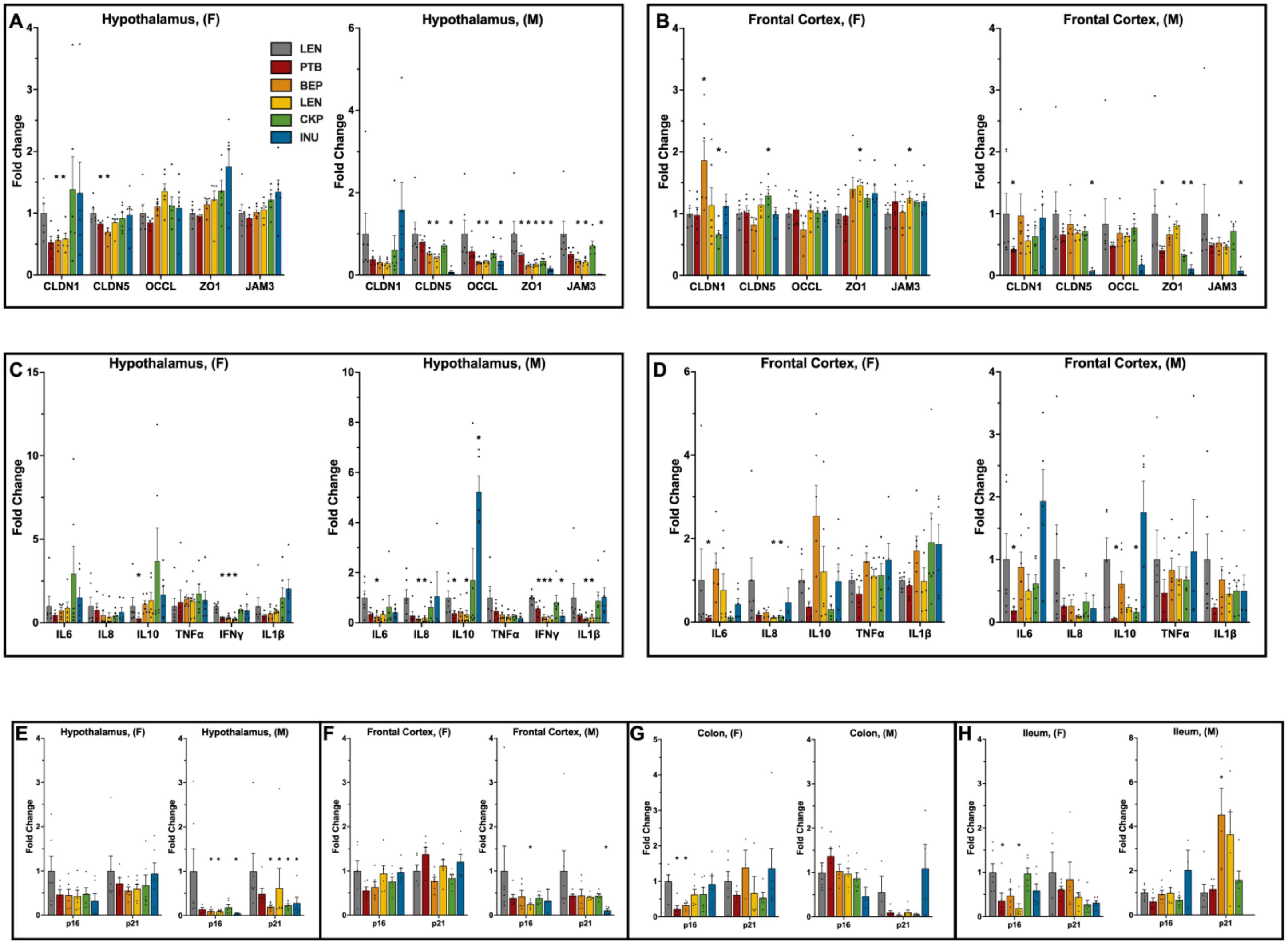

Supplementary Figure S4. Sex-specific effects of RS and inulin supplementation on the serum endocrine levels of (**A)** Leptin, **(B)** Insulin, (**C)** Glucagon, **(D)** Lipid profiles (females), and **(E)** Lipid profile (males). RS and inulin supplementation effects on hepatic function and glucose levels in (**F)** Combined, **(G)** Females, and (**H)** Males. *p<0.05

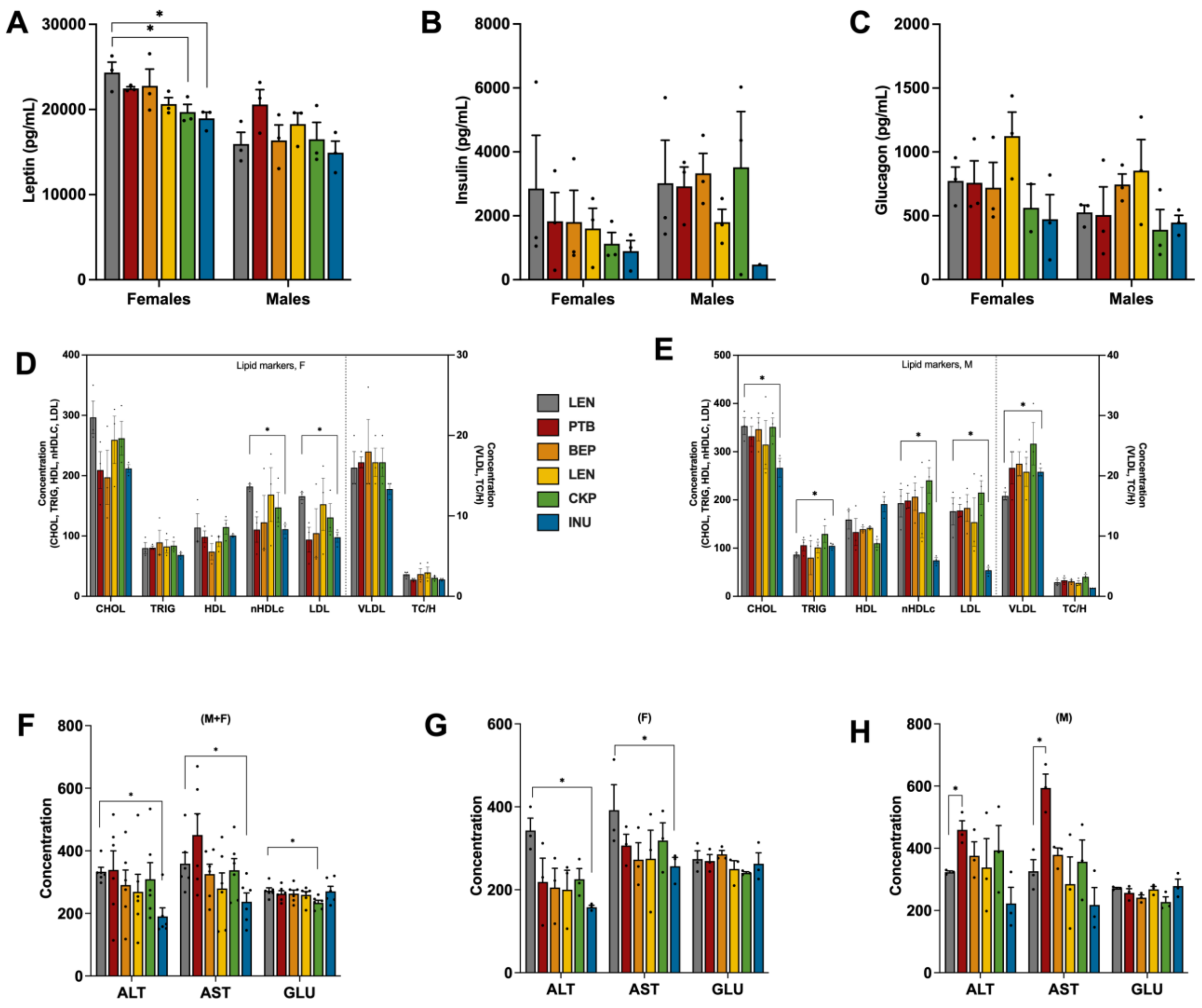

Supplementary Figure S5. Heatmap showing associations between gene expression levels of inflammatory markers and tight junction proteins in the hypothalamus and frontal cortex regions of brain with gut microbiome and metabolite fingerprints. Black/white dot within a cell indicates. ●$p<0.05$, adjusted with Benjamini-Hochberg correction

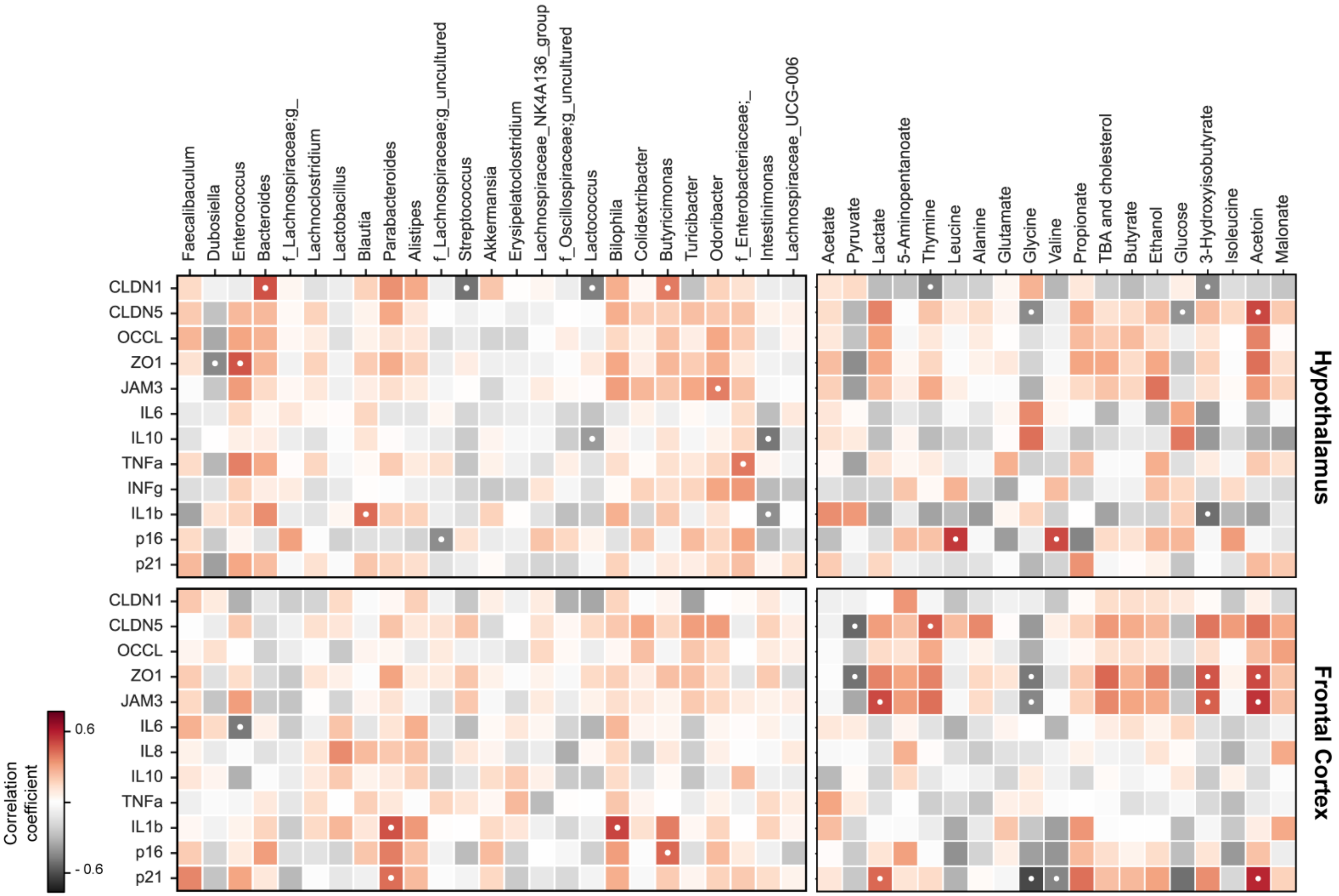

Supplement: Supplementary file 1 [file Data_Sheet_1.PDF]
